# Combined Bleaching, Microabrasion and Resin Infiltration for Postorthodontic White Spot Lesions: A Case Report

**DOI:** 10.1155/crid/4629346

**Published:** 2026-10-10

**Authors:** Sabah Almutairi

**Affiliations:** ^1^ Department of Conservative Dental Science College of Dentistry Qassim University Buraydah Saudi Arabia qu.edu.sa

**Keywords:** enamel microabrasion, in-office bleaching, postorthodontic, resin infiltration, white spot lesions

## Abstract

White spot lesions (WSLs) are a form of subsurface enamel demineralization frequently observed following orthodontic treatment and adversely affect dental aesthetics. This case report describes the successful management of noncavitated postorthodontic WSLs in the maxillary anterior teeth of a 32‐year‐old female. A sequential, minimally invasive approach was utilized, combining in‐office bleaching, microabrasion, and resin infiltration (Icon, DMG, Hamburg, Germany). The protocol led to notable aesthetic improvement and satisfactory shade integration across two clinical sessions. In this patient, resin infiltration combined with microabrasion provided an effective treatment modality for postorthodontic WSLs.

## 1. Introduction

White spot lesions (WSLs) are a type of incipient enamel caries characterized by subsurface enamel demineralization that originates from sustained plaque retention around fixed appliances. Acid‐producing bacteria within the plaque metabolize dietary sugars, releasing organic acids that cause subsurface enamel demineralization. As this process unfolds beneath a relatively preserved outer enamel surface, increased porosity alters light scattering through the tissue. The refractive index (RI), a measure of the varying transparency or translucency of demineralized enamel, is lower than that of intact or sound enamel, resulting in a white or opaque appearance often described as chalky or “whitish” [1]. Increased risk of WSL is a direct result of orthodontic treatments, as the pupils of these treatments often retain plaque build‐up that gathers around the braces. Reports of WSL incidence postdebonding range from ~15% to > 90%, depending on the level of oral hygiene maintained and the duration of orthodontic treatment [2]. Many WSLs, if left untreated, persist and worsen over time. These untreated WSLs can significantly compromise the patient′s aesthetics [3].

The traditional management of WSLs is the use of fluoride and remineralizing pastes. These products, while halting lesion progression, do not markedly or fully improve enamel translucency. With routine microabrasion, treatment regimens remove superficial lesions but also irreparably remove 100–200 *μ*m of enamel [4].

The use of microabrasion is very different than what has been categorized as enamel resin infiltration. Resin infiltration is a “minimally invasive” means of treating WSL and is the most aesthetically acceptable option, as it uses a light‐cured resin (and is more than likely to contain other materials that cross‐link and polymerize) to penetrate the pores of the enamel lesions. The WSL that is infiltrated is no longer detectable at a distance, as it has altered its optical properties and now resembles those of the adjacent sound tooth structure. Additionally, as stated, it is more advanced because it does not involve any tooth drilling (only acid etching) to complete the process [5].

To manage superficial enamel defects, a minimally invasive approach is widely favored to preserve as much healthy tooth structure as possible while restoring aesthetics and function. Although techniques such as enamel microabrasion involve a controlled physical loss of tissue of approximately 100–200 *μ*m, combining them with resin infiltration provides a minimally invasive alternative that avoids traditional operative cavity preparations [6]. Many studies have documented and demonstrated that Icon resin infiltration removes economic and, more importantly, aesthetically pleasing WSLs. Most of these documented cases have been completed, and studies have been conducted over 6–24 months, with some extending to longer periods [6, 7].

The present case highlights the clinical application of a sequential minimally invasive treatment protocol rather than a novel treatment modality. Although bleaching, microabrasion, and resin infiltration have each been described individually in the literature, this report demonstrates how their careful combination and sequencing can effectively improve the aesthetic appearance of postorthodontic noncavitated WSLs within a two‐clinical‐visit protocol. This case report may help clinicians by providing a reference for the detailed treatment protocol followed and its positive outcome.

### 1.1. Key Clinical Message

A 32‐year‐old female patient presented with noncavitated WSL lesions on the maxillary anteriors postorthodontic treatment. Treatment using in‐office bleaching, microabrasion, and resin infiltration, in accordance with the principles of minimally invasive dentistry, led to improved aesthetics, with a significant reduction in lesion opacity and better integration with the surrounding enamel.

## 2. Case Presentation

### 2.1. History and Examination

A 32‐year‐old female patient presented to the University dental clinic, expressing concern about WSLs on her upper anterior teeth. Two years ago, she completed her fixed‐bracket treatment. During intraoral examination, there were six scattered, opaque, and chalky lesions on the facial surfaces of her maxillary incisors and canines (Teeth #13, 12, 11, 21, 22, and 23). Some of the lesions presented with smooth, round, and noncavitated surfaces with intact enamel, classified as ICDAS (International Caries Detection and Assessment System) Code 1 (first visual change in enamel visible only after air‐drying) and Code 2 (distinct visual change in enamel visible even when wet). The lesions presented were nontender and aesthetically displeasing. The radiographs showed no underlying radiolucencies, consistent with superficial enamel lesions. The remaining dentition was examined; no caries was present, and there was a consistent and routine dental treatment history, with the only exception of poor orthodontic dental hygiene. Clinical photographs were obtained using a Canon EOS 80D digital SLR camera, equipped with a 100 mm f/2.8 Macro lens and twin flash. The camera settings were standardized for all sessions: aperture f/22, shutter speed 1/125 s, and ISO 100. To ensure color consistency, the white balance and exposure settings were kept constant across all photographic sessions, and no digital color alterations were performed (Figure 1).

**Figure 1:**
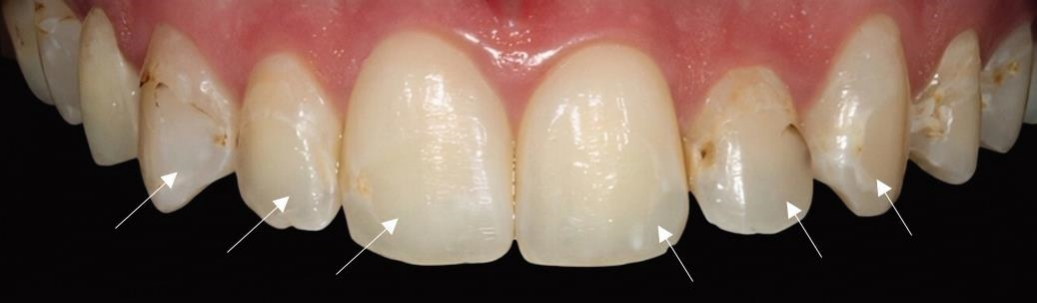
Postorthodontic WSLs.

### 2.2. Diagnosis, Investigations, and Treatment

The postorthodontic WSLs (hypomineralized enamel) were diagnosed based on the study findings, including a history of orthodontic treatment and smooth‐edged lesions. These lesions develop from prolonged biofilm accumulation around orthodontic attachments, in which acid produced by cariogenic bacteria causes repeated episodes of enamel demineralization. The biochemical changes at the enamel surface lead to subsurface mineral loss, whereas the outer layer of the enamel generally remains intact, giving these lesions their characteristic opaque white appearance.

This patient was moderately at risk of developing dental caries. Earlier WSL presence had suggested a risk due to poor hygiene. There was normal salivary flow of 1.2 mL/min. A brief dietary assessment via a 3‐day food diary revealed a low frequency of sugar intake and no acid challenge from the diet. Nevertheless, she demonstrated inadequate plaque control during orthodontic treatment, which is one of the risk factors for WSLs. Her baseline caries assessment, DMFT = 2 (consisting of two decayed teeth, zero missing/filled teeth), was classified as low. Among the various risk factors for fixed appliances, plaque and sugar consumption, only diet, and oral hygiene were considered.

All teeth were professionally cleaned prophylactically with pumice paste to enhance resin penetration, prevent surface contaminants, and remove surface pellicle, plaque, and debris. Topical fluoride was avoided. To optimize the overall aesthetic color blend, a brief in‐office bleaching session was initiated. Bleaching reduces the color contrast between the WSLs and the surrounding natural enamel. The bleaching procedure was performed using a chemically activated 35% hydrogen peroxide gel (Opalescence Boost, Ultradent Products, South Jordan, United States). The gel was applied directly to the facial surfaces of the teeth in two 20‐min cycles, for a total active treatment time of 40 min. No light activation or laser was utilized during the procedure. Immediately after rinsing the bleaching gel, a desensitizing gel containing 3% potassium nitrate and 0.25% sodium fluoride (UltraEZ, Ultradent Products) was applied for 15 min to prevent postoperative sensitivity (Figure 2).

**Figure 2:**
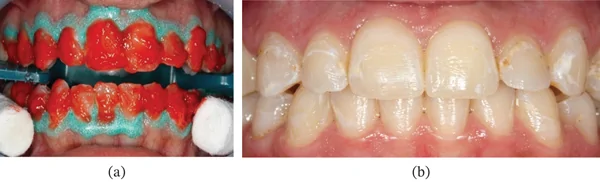
In‐office bleaching. (a) Bleaching, (b) Post‐bleaching.

Following a 7‐day postbleaching interval, microabrasion was performed with a pumice and a 6.6% hydrochloric acid slurry (PREMA, Premier Dental) to mechanically remove the superficial, oxygen‐rich enamel layer and address potential oxygen inhibition. This facilitated proper resin penetration and sealing during the same visit. A rubber dam was used to isolate the arches, and interproximal and labial floss ligatures were used to secure the dam. Rubber dam isolation is critical when using hydrochloric acid to protect soft tissues (Figure 3).

**Figure 3:**
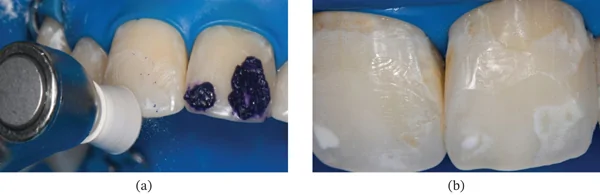
Microabrasion. (a) During microabrasion, (b) after microabrasion with approximate magnification ×2.

The decision to use both microabrasion and resin infiltration depended on the age of the lesions. Since the patient completed orthodontic treatment 2 years prior, the WSLs had matured and become inactive, forming a hard, highly mineralized outer surface due to long‐term exposure to saliva. Standard etching with 15% hydrochloric acid alone is often insufficient to open these old, consolidated lesions. Therefore, microabrasion was used first as a minimally invasive method to remove this resistant surface layer and clear surface stains, creating a receptive base for the resin to fully penetrate the enamel body.

The Icon (DMG, Hamburg, Germany) Etch Kit was used to treat the enamel surface with WSLs. Etching was performed for 2 min per lesion using microbrush agitation, as per the manufacturer′s instructions, with Icon Etch 15% hydrochloric acid gel for enamel lesions. After a 30‐s water rinse, the enamel appeared frosted white. A second 2‐min etch was performed on any thick lesions to maximize porosity, as per the manufacturer′s instructions regarding thick lesions, and on any remaining visible opacities. A thorough rinse and gentle air‐drying were performed after the etch until the areas were chalky and clean. Alcohol drying was done with a microbrush (Figure 4a).

**Figure 4:**
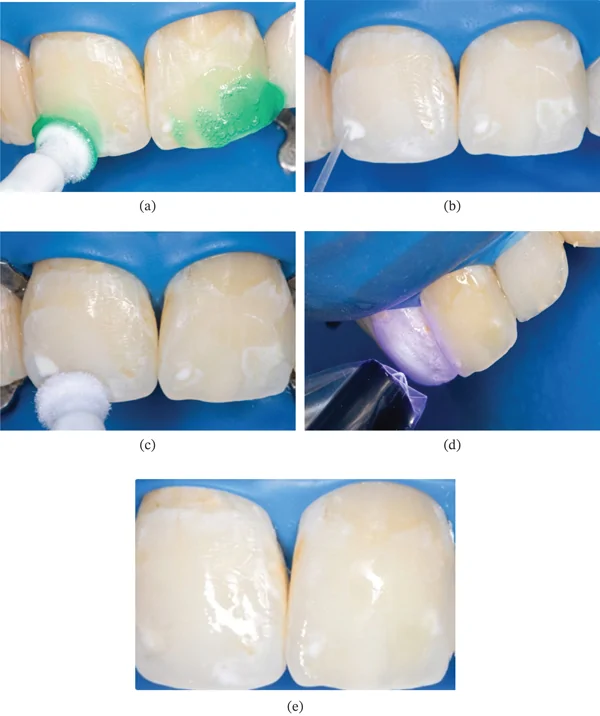
Icon resin application. (a) Acid etching, (b) drying with ethanol, (c) resin infiltrant application, (d) light curing, (e) after resin infiltration.

Icon Dry (99% ethanol) was applied to each lesion for 30 s, then dried with oil‐free air. In this step, the moisture is removed from the enamel, further widening the open pores and showing the expected appearance after resin infiltration. When ethanol was applied, the teeth appeared slightly more translucent, indicating that the demineralization was sufficient. This was a preview of the outcome (Figure 4b).

Each lesion was treated with the low‐viscosity infiltrant resin (Icon infiltrant, primarily TEGDMA). An ample amount of resin was submerged over the etched enamel and stirred for 3 min to facilitate deep penetration into the substrate pores. Additionally, a microbrush was used to enhance resin wicking into the lesion. Cotton rolls were used after 3 min to remove excess resin, and a gentle airflow was applied. Then, a high‐intensity LED curing light was used for 40 s (40 s for each labial side) to cure the infiltrant on each tooth (Figure 4c,d).

For each lesion, a second coat of Icon infiltrant was applied for 1 min to address any remaining microporosities, after which excess was again cleaned, and the resin was cured for 40 s. At this point, glycerine gel was applied to the top of the lesion, and a final 20‐s cure was performed to eliminate an oxygen‐inhibited layer and ensure complete polymerization. This two‐step infiltration (3 + 1 min) is within the protocol suggested by DMG.

Once the rubber dam was removed, the gingival margins were assessed. A sharp scaler was used to remove excess resin from the gingival margins. The final finishing and polishing were completed using a sequential, three‐step protocol to achieve a natural, high‐gloss enamel surface. First, medium and fine aluminum oxide polishing discs (Sof‐Lex, 3 M ESPE) were used to remove any excess resin from the margins gently; coarse discs were intentionally avoided to protect the fresh, thin layer of cured resin from excessive abrasion. Next, flexible silicone‐impregnated polishers (Enhance, Dentsply Sirona) were used to smooth the facial contours and curved transitional line angles of the anterior teeth. Finally, a high‐gloss luster was achieved by applying a diamond polishing paste (DiaShine, Intraoral HP) with a soft felt wheel. This combined sequence ensured a smooth, plaque‐resistant surface that perfectly mimicked the natural wet look of healthy enamel.

There was a noticeable smoothness of the teeth that looked similar to the enamel. The entire procedure was performed without local anesthetic. The duration of the process was 45 min. Patient aesthetic satisfaction was evaluated immediately after treatment using a visual analog scale (VAS) for aesthetic satisfaction ranging from 0 to 10 (where 0 represented “completely dissatisfied” and 10 represented “completely satisfied”). The patient marked their experience on the line, and scores were recorded for analysis. The patient was advised to keep her oral cavity clean and was prescribed fluoride toothpaste (1450 ppm) (Figure 4e).

### 2.3. Results

In the present case study, there was a significant improvement in postorthodontic WSLs on the maxillary anterior teeth following minimally invasive treatment with resin infiltration. Improvement in clinical results was evaluated using parameters such as lesion extent, surface characteristics, integration with surrounding enamel, and patient opinion. There was a marked difference in the appearance of WSLs right after the procedure. The previously opaque areas of enamel became translucent and seemed to integrate with the surrounding enamel (Figure 5).

**Figure 5:**
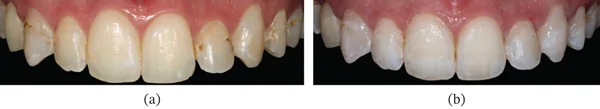
Treatment of postorthodontic WSL. (a) Pretreatment of WSLs, (b) immediate post‐treatment of WSLs.

Following treatment, a marked aesthetic improvement was observed in the postorthodontic WSLs on the maxillary anterior teeth (central incisors, lateral incisors, and canines). Tactile evaluation with a dental explorer revealed smooth, intact enamel surfaces with no detectable roughness, voids, or surface flaws. Immediately posttreatment, the patient′s subjective aesthetic feedback was recorded using a standard VAS, on which she rated her aesthetic satisfaction as a 9 out of 10. The overall timeline and treatment protocol are summarized in Table 1.

**Table 1:** The timeline of the patient′s presentation, diagnostic assessment, minimally invasive treatment sequence (bleaching, microabrasion, and resin infiltration), and immediate clinical outcomes following management of postorthodontic WSLs.

Time	Clinical event	Findings/intervention
2 years before the presentation	Completion of fixed orthodontic treatment	Patient completed orthodontic treatment; inadequate oral hygiene during treatment predisposed to white spot lesions (WSLs).
Presentation	Chief complaint	32‐year‐old female presented with an aesthetic concern regarding white spot lesions on maxillary anterior teeth.
Initial examination	Clinical assessment	6 distinct noncavitated, chalky white lesions on Teeth #13, 12, 11, 21, 22, and 23 (ICDAS 1–2); lesions are nontender.
Investigations	Diagnostic work‐up	Radiographs showed no dentinal involvement or radiolucency; DMFT = 2; normal salivary flow; moderate caries risk assessment.
Treatment planning	Diagnosis	6 distinct postorthodontic noncavitated white spot lesions on Teeth #13, 12, 11, 21, 22, and 23. Decision for minimally invasive aesthetic management.
Visit 1	Prophylaxis	Professional cleaning with pumice; fluoride application withheld.
Visit 1	In‐office bleaching	35% hydrogen peroxide (Opalescence Boost) is applied to reduce lesion contrast.
Visit 2	Microabrasion	6.6% hydrochloric acid–pumice slurry (PREMA) under rubber dam isolation.
Visit 2	Resin infiltration	Icon protocol: 15% hydrochloric acid etching, ethanol drying, resin infiltration (3 + 1 min), light curing, finishing and polishing. Total procedure time ≈45 min.
Immediately after treatment	Clinical outcome	White spot lesions became less opaque and blended with surrounding enamel; a smooth enamel surface was achieved. Excellent immediate stable aesthetics achieved, patient highly satisfied with treatment. No follow‐up was obtained as the patient did not return for recall.

### 2.4. Patient Perspective

The patient felt self‐conscious about her smile due to the white spots. Immediately after treatment, she expressed great satisfaction with the aesthetic outcome, noting a significant boost in her self‐esteem and quality of life, with no sensitivity.

## 3. Discussion

Performing bleaching before microabrasion and resin infiltration unifies the background tooth shade, allowing a more precise evaluation of the remaining lesion. A 1‐week waiting interval was observed after bleaching before proceeding with the microabrasion and resin infiltration session. Furthermore, it is hypothesized that microabrasion may help remove the outermost, oxygen‐rich enamel layer resulting from bleaching, thereby reducing oxygen inhibition and facilitating resin infiltration and polymerization [8]. However, this rationale serves as a clinical hypothesis rather than a confirmed mechanism within a single case report.

Resin infiltration enhances the aesthetic appearance by filling the lesions with a resin having an RI of 1.46–1.52, thereby replacing the previously present low‐RI constituents (air and water). The patient′s aesthetic objectives were met after one Icon infiltration appointment, with a remarkable reduction in white spots. Furthermore, the combined protocol of microabrasion and resin infiltration offers a minimally invasive alternative that effectively masks lesions while preserving the bulk of the enamel structure without the need for dental handpieces. Resin infiltration addresses both the structural and optical aspects of WSLs, and the basic concept of resin infiltration is to replace the air and water in porous enamel lesions with a low‐viscosity resin that has an RI similar to that of sound enamel [9]. Enamel free of caries has an RI of about 1.62. Demineralized enamel, on the other hand, has air (RI ≈1.0) or water (RI ≈1.33), which results in light scattering and the white appearance [10]. The infiltrant resin in question (Icon) has an RI of approximately 1.46, thereby enhancing translucency and reducing light scattering [11].

In the present case, the use of 15% hydrochloric acid (Icon Etch) was unavoidable to remove the hypermineralized surface layer and allow deep resin penetration. Hydrochloric acid etching has been shown in several studies to be more effective than phosphoric acid at opening enamel pores, allowing infiltration [12]. The evidence indicating that multiple etching cycles improved penetration depth and provided better aesthetics justified the choice of repeated etching cycles for more resistant lesions [13]. The literature further supports the outcomes. Studies have shown that resin infiltration yields better immediate aesthetic improvements in WSLs than topical fluoride or bioactive agents and results similar to or better than those of enamel microabrasion, with much less enamel removal. It was learned in this case that a single well‐executed infiltration with extended penetration time achieved clinically stable color improvement for up to 6 months, whereas studies have reported stable masking effects lasting 1–2 years postinfiltration [14].

On the other hand, microabrasion is characterized by multiple cycles and a permanent reduction in enamel thickness, while remineralizing pastes still leave lesions visible. This particular case has several key technical factors that ensure its success. Cleansing and isolation are essential procedures; the former removes any barriers to resin permeation, whereas isolation with a rubber dam keeps the gum away from the acid (15% hydrochloric acid is caustic). All etch‐and‐rinse cycles recommended by the producer and the literature (15% hydrochloric acid etching ×2, ethanol drying, 3 resin soaks +1 etch cycle) for the infiltration technique were performed. Moreover, an additional etch cycle was done for some lesions to increase their porosity [14]. In the literature, the following has been documented regarding resin uptake: it is best achieved in deep sites with several etching sessions. Curing for 40 s per surface is necessary to ensure proper polymerization of the resin relative to the infiltrant [15].

However, there are certain clinical considerations to take into account when using the resin infiltration treatment approach. This therapy is strictly recommended for use on noncavitated lesions only (ICDAS 1‐2); if a tooth cavity occurs, a shift to traditional dental restoration should be made. The resin infiltration method does not completely hide the defect in cases of deep enamel discoloration or opacity, such as that caused by dental fluorosis. Another limitation of this procedure is the risk of discoloration of the resin matrix from extrinsic stains if the patient smokes heavily and drinks a lot of coffee. Thus, the patient′s high level of compliance and good oral hygiene are required. In our case, a short‐term fluoride varnish treatment protocol was recommended along with the introduction of strict oral hygiene measures. The reason for this recommendation was to control these factors and protect the aesthetic effect [15, 16]. It is also necessary to point out that the study′s outcome should be considered in the context of other limitations, such as the inclusion of only one patient and the lack of long‐term follow‐up, as the patient was lost to recall after the active treatment phase. Visual improvement was evaluated subjectively, without objective color or fluorescence evaluation or image analysis. Therefore, the results cannot be generalized, and prospective studies with standardized outcome measures are warranted.

A limitation of this report is that combining in‐office bleaching, enamel microabrasion, and resin infiltration in a sequential protocol precludes isolating the individual contributions of each procedure to lesion masking and shade enhancement. Although this multimodal approach achieved a synergistic aesthetic outcome, controlled clinical studies comparing each procedure individually with the combined treatment protocol are required to quantify their individual clinical effects.

Although resin infiltration is an established method for treating noncavitated white spots, few articles discuss its use alongside other minimally invasive treatments, such as in‐office bleaching and enamel microabrasion. In the current study, the application of the said minimally invasive techniques in a well‐thought‐out sequence led to aesthetic enhancement without compromising the tooth structure through restorative therapy. The rationale for the treatment procedure and the outcome will provide useful information to assist practitioners in dealing with similar cases in their day‐to‐day dental practice. Nevertheless, the favorable outcome observed in this patient suggests that this minimally invasive treatment strategy can be a predictable and effective option for appropriately selected cases.

## 4. Conclusion

In conclusion, this case demonstrates that a sequential, minimally invasive protocol—combining bleaching, microabrasion, and resin infiltration—yielded a favorable immediate aesthetic outcome for noncavitated postorthodontic WSLs in this patient. The treated lesions became less noticeable and blended naturally with the surrounding enamel, leading to high patient satisfaction. However, as these findings are limited to a single case report without long‐term follow‐up, further clinical studies are needed to evaluate the long‐term stability and broader predictability of this approach.

## Author Contributions

conceptualization, methodology, data curation, and writing—original draft preparation, reviewing and editing.

## Funding

No funding was received for this manuscript.

## Ethics Statement

In accordance with institutional policy, ethics committee approval is not required for a single‐patient case report.

## Consent

Informed written consent was obtained from the patient to undergo the procedure, and consent was also obtained for the publication of her deidentified data and images.

## Conflicts of Interest

The author declares no conflicts of interest.

## Supporting information


**Supporting Information** Additional supporting information can be found online in the Supporting Information section. File S1: CARE checklist.

## Data Availability

The data that support the findings of this study are available from the corresponding author upon reasonable request.
